# Health Risk in Urbanizing Regions: Examining the Nexus of Infrastructure, Hygiene and Health in Tashkent Province, Uzbekistan

**DOI:** 10.3390/ijerph15112578

**Published:** 2018-11-18

**Authors:** Saravanan Veluswami Subramanian, Min Jung Cho, Fotima Mukhitdinova

**Affiliations:** 1 Center for Development Research, University of Bonn, 53113 Bonn, Germany; 2 Faculty Governance and Global Affairs, Leiden University College the Hague, 2595 DG Den Haag, The Netherlands; m.j.cho@luc.leidenuniv.nl; 3 Scientific-Research Institute of Public Health and Healthcare Organization, Ministry of Health of the Republic of Uzbekistan, 100011 Tashkent, Uzbekistan; fotima.azimova777@gmail.com

**Keywords:** urban health, infectious diseases, health risk assessment, Central Asia, Uzbekistan

## Abstract

Worldwide, development agencies have increased their investments in water supply and sanitation as a “powerful preventive medicine” to address infectious diseases. These interventions have focused on on-site technical interventions or social engineering approaches, emulating the result-based targets of the development goals. Against this backdrop, the study examines the following research question: What is the role of socio-cultural backgrounds, housing characteristics, and environmental hygiene practices in addressing water-transmitted diseases in the Tashkent province of Uzbekistan. In a country where public statistics and official maps are rarely accessible, and research is restrictive, the study carried out a household survey using open data kit (ODK) between July and October 2015 in Olmalik, an industrial district, and the Kibray urbanizing district in the province. The findings reveal that demographic factors, poor sanitation practices, housing characteristics, and social behaviors are key predictors of water-transmitted diseases in the two districts. In the industrial township, poor housing, larger household size, and poor excreta disposal habits increased the occurrence of diseases, while in urbanizing districts, higher household size, frequently eating out, and access to public taps significantly increased the occurrence of water-transmitted diseases. The study, which was carried out in a challenging institutional environment, highlights the need for Uzbekistan to focus their policies on environmental hygiene, demographic factors and social behavior as key interventions rather than merely on on-site drinking water and sanitation interventions.

## 1. Introduction

The World Health Organization claims that health is a vital investment in Central Asia [1]. The region experiences serious difficulties in providing consistent surveillance data on emerging diseases, or even on classic water-related diseases. Hence, public health systems in Central Asia are in a critical state. According to the data that are available, Central Asia appears to suffer from a high burden of water-transmitted diseases [2]. Advances in science and technology and political change have shaped these countries and their public health systems. Following the collapse of the Soviet Union in the 1990s, the Central Asian countries began a transition from centrally planned economies towards market-oriented economic development, characterized by better environmental protection and healthcare provision. Before 1991, water provision and healthcare followed the Soviet model, which focused on the centralized regulation of the water supply, sewage, and hygiene control to combat water-transmitted diseases. The infectious agents are spread by the fecal–oral route, in which water may play an intermediate role [3]. These are termed as water-transmitted diseases (WTDs) in the paper, which broadly refers to water-borne, water-related, water-based and water-washed diseases [3]. After gaining their independence, the Central Asian countries faced the problem of organizing basic infrastructure and healthcare services in the face of a legacy of inadequate investment in public water systems, poor institutional arrangements, and information gaps related to preventive action. The proportionally high disease burden in the Central Asian region, and especially the high infant mortality rates due to diarrheal diseases, put extraordinary stress on health systems to not only provide surveillance but also outbreak detection and management.

Uzbekistan has made significant attempts to reform its drinking water sanitation and healthcare systems. Indeed, the country has invested substantially in upgrading its water supply and sanitation services in recent years. From 1995 to 2014, Uzbekistan’s total borrowing for improvements to these services amounted to US$ 344.1 million, the highest total for any country in Central Asia [4]. These interventions have largely focused on on-site technical interventions or social engineering approaches, emulating the result-based targets of the development goals [5]. Technocentric solutions (e.g., on-site drinking water and sanitation, technologies for in-house water treatment or sanitation, vaccinations, drugs, and mosquito nets) and socially engineered solutions (through awareness generation and public participation) have been widely promoted as powerful preventive medicines at the individual/household level [6]. The result of these investments is poor water quality as over half of the population do not have access to piped water and a public health system [7,8]. The WHO reports that diarrhea is the leading cause of death among children under 5 with Uzbekistan reporting about 10% and about 800 children reporting severe gastroenteritis annually. Semenza et al. [9], through a combination of active diarrheal surveillance and a randomized intervention study, revealed that poor water quality contributes to a substantial diarrheal burden among the population of the Nukus region. They argued that cross-contamination in the municipal water supply due to leaky pipes is the major cause of the diarrheal burden in the region. Through a controlled prospective cohort study among children in rural Uzbek, Gungoren et al. [10] demonstrated the effectiveness of hygiene promotion in reducing the risk of intestinal parasite reinfection. Sharapova et al. [11] used surveillance information to assess the etiology of acute viral hepatitis in Uzbekistan. Herbst et al. [12] investigated the risk factors water, sanitation, and hygiene in terms of diarrheal diseases among children, finding that unhealthy excreta handling and disposal habits along with unsafe drinking water treatment and handling practices have a significant role in the fecal–oral disease transmission route in the Khorezm province in Uzbekistan. However, the quality of water is worsened by pollution from domestic waste, organic matter, mineral fertilizers, pesticides, and sewers, which has a considerable effect on human health [13]. Furthermore, industries in Uzbekistan withdraw 1.2 km^3^ of water annually, almost half of which is returned as industrial wastewater, posing a serious threat to the quality of the country’s drinking water [14]. According to Charyev et al. [15], about 2600 medium small businesses operate with water intake or outtake of the open reservoirs. These macro-level clinical studies highlight the need for a more in-depth understanding of the causes of infectious diseases. The inadequate availability of statistics, restricted research environments, and insufficient application of methodological tools significantly hamper a comprehensive social, environmental, and hygiene assessment of WTDs at the household scale. Such assessment would help to effectively target interventions. This paper calls for an understanding of the social, economic, and environmental factors behind the occurrence of WTDs in two districts in the Tashkent province [6,7,8,9,10,14]. Specifically, the objective of this study is to examine the socio-cultural backgrounds, drinking water and sanitation, housing characteristics, and hygiene practices and their role in influencing WTDs among households in two urbanizing districts in Uzbekistan.

This paper is organized in five sections. The following section provides the backdrop of the case study districts. The methodology is detailed in Section 3. The findings are presented in Section 4. The following discussion section highlights the significant role of demographics, housing characteristics, and hygiene practices and their impact on WTDs in urbanizing districts. It further elaborates the challenges of field research in this part of the world. The final concluding section highlights the policy implications from this study which calls for technical interventions to be surpassed in order to address infectious diseases.

## 2. Study Background

**Figure 1:**
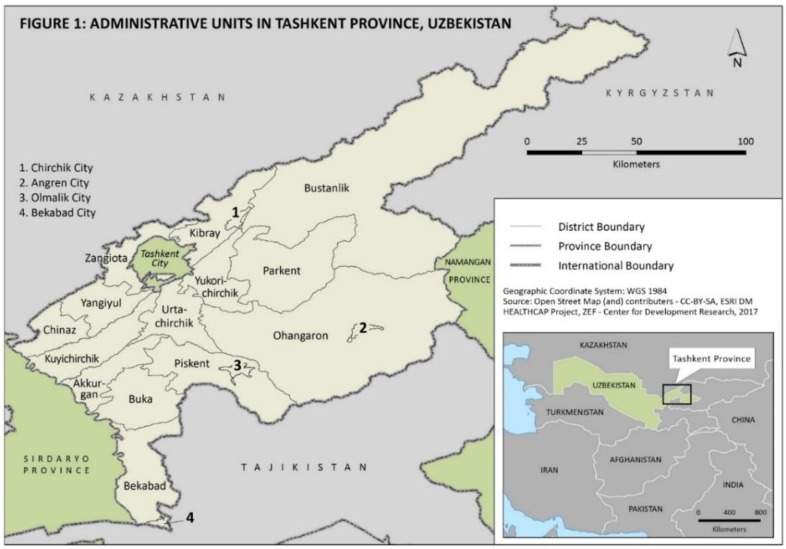
Administrative units in Tashkent province, Uzbekistan.

The Tashkent province is in the northeastern part of the country, between the Syr Darya River and the Tien Shan Mountains. As of 2014, the population was 2.6 million living in 15,300 Km^2^. The province consists of four towns and 15 districts, excluding Tashkent City, which is governed as an independent administrative unit (Figure 1). The climate of the province is moderately continental with vertical zonation. It has high daily and annual fluctuations in temperatures and annual rainfall. Despite its proximity to Tashkent City, only about 82% and 70% of the population (as on 2005) are provided with piped water supply and access to sewage systems, respectively. The province is faced with a pollution threat due to urbanization and industrialization, while its rural areas suffer from a lack of wastewater treatment and sewage plants. This has a significant impact on human health. A spatial temporal analysis in the province revealed that the province is vulnerable to four major water-borne diseases: enterobiasis, hepatitis A, acute intestinal infection, and dysentery [16]. The incidence rates for enterobiasis were found to be highest, with a four-year (2011–2014) average of about 1084 cases per 100,000 population; the lowest were for dysentery, with an average of 28 cases per 100,000 population. The prominent districts in the province are Akkurgam Zangiota, Bekabad, Ohangaron, Kibray, and. Among the cities, Olmalik reported a high incidence of enterobiasis (about 1000 cases) and hepatitis A (about 250 cases), while Angren reported a high incidence of hepatitis A (about 170 cases), acute intestinal infections (about 350 cases), and dysentery (about 55 cases).

To examine the socio-cultural behaviors, housing characteristics, and environmental hygiene factors and their effect on WTDs, the Olmalik and Kibray districts were selected for the study. The Olmalik district is located close to Tashkent City and is predominantly an industrial region. The discovery of a copper deposit in Olmalik led to rapid growth in the urban population beginning in 1954. The district has the largest cluster of non-ferrous metallurgy mining plants in the country, namely, copper concentrators, lead–zinc plants, and sulfuric acid production facilities. The district’s poor sewage network as well as industrial sulfur fumes have been major issues in the environmental cleanup in Olmalik [17].

The facilities for industrial wastewater treatment are offsite, and there are no effective local wastewater treatment plants. Studies [18,19] have shown that nitrate, sulfate, and ammonium exceeded recommended maximum allowable concentration (MAC) values for some water samples from the Chirchick (Appendix A) River due to discharges from the agricultural sector and other industries. The major sources of pollutants in the Chirchik and Akhangaran Rivers suggest that the existing water treatment technologies are ineffective and obsolete, which contributes to contaminated industrial wastewater [18,19]. Although the district receives water from the two rivers (Chirchik and Akhangaran), the ground water drawn from the wells along the Akhangaran river remains the main water source. In this district, Olmalik city was selected for study as it reported the highest incidence of WTDs in the province between 2011 and 2014 [16]. The average incidence rates of acute intestinal infections and hepatitis A were about 296 and 250, respectively, between 2011 and 2014. Second, its industrial composition plays a key role in the socio-economic and hygiene characteristics affecting the health of the people [19].

The Kibray district is located adjacent to Tashkent City and contributes to the province’s economic growth through its agro-industrial and textile industries. The district has about 186,600 residents, who are largely of Uzbek ethnicity. The district has a mix of rural and urban characteristics, probably due to its proximity to Tashkent City. In 2014, about 54% of the population were rural and the rest urban. The economy includes a mix of industries, agriculture, and education and service sectors, which is a typical characteristic of urbanizing regions. The education and service sector is responsible for over 22% of the employment in the district. The access to drinking water increased from 83% in 2008 to 92% in 2013. However, only 23% of the households have access to the sewer system. The rest dispose domestic wastewater in open drains or agriculture fields. The Asian Development Bank (ADB) loan in 2016 aimed to modernize the *vodokanals* (water supply and sanitation agencies) by upgrading the existing water supply networks, providing portable water treatment services, rehabilitating the water distribution network and improving its commercial and systems management [20]. Due to the lack of a district map, the Kibray central region was selected for the survey, as it exhibited a peri-urban characteristic, with administrative offices and residential areas combined with an agriculture-related economy.

## 3. Methods

The study was carried out using open data kit (ODK) in two districts of the Tashkent province. The availability of public statistics in Uzbekistan was a challenge, as district-level information is rarely available in the public domain. It is only since 2015 that state-level population data as well as other social indicator data have been openly available (national statistics homepage https://stat.uz, https://data.gov.uz/, as well as the Tashkent province homepage www.toshvil.uz). However, province-level data are not easily accessible, nor is the official map of the districts. The inadequate availability of public statistics on the demographic, socio-economic, and health status of the population in the country hindered the selection of field sites and the stratification of the population for the household survey. Following Kondo et al. [21], the paper applied a simple random spatial sample using geographic information systems (GIS) and global positioning systems (GPS) to select households. This method was selected due to the lack of access to household information in Uzbekistan [22]. Although random probability sampling is the golden standard, simple random spatial sampling has been adopted as an alternative when statistics and household listings are not readily available [21,23,24].

The questionnaire was prepared in discussion with officials in the Ministry of Health, Centre for Sanitary Epidemiology Surveillance, Republican Institute for Sanitary, Hygiene and Occupational Diseases, and Research Institute for Irrigation and Water Problems and tested before operationalizing in the field. The questionnaire consisted of sections pertaining to basic facilities in the households, socio-demographic characteristics of the families, hygiene practices, migration history, health history (WTDs in the last six months and chronic diseases in the last one year), and health-seeking strategies. WTDs refer to gastroenteritis, acute diarrhea, bacillary dysentery, viral hepatitis, and enteric fever.

Once the questionnaire was finalized, cross-sectional surveys were conducted using Open Data Kit (ODK) software (opendatakit.org) on android tablets and smartphones [25]. ODK is an open-source suite with user-friendly web-interfaces for designing survey forms. We used ODK for several reasons. (1) ODK is run by the Android operating system and runs on the widest selection of smartphones/tablets with minimal costs [26]. (2) ODK is open-access and freely available on the market and displays these to users on mobile devices running Android. (3) ODK supports various types of questions including text entry, multiple choice, and check boxes as well as GPS location information and photos both online and offline. Data were collected using 7″ low cost smartphones with an Android 4.1.1 operating system. ODK was used locally to program the questionnaire with inbuilt logical skips and range checks. Data were downloaded weekly from the smartphones and imported into a backed-up Microsoft Access database. Participant confidentiality was maintained with the use of password-protected tablets and encrypted databases. Since its introduction, ODK has been widely used for electronic data capture in resource-limited settings such as Malawi, Kenya, and Nepal among many others [27,28,29]. 

The survey was carried out between July and October 2015, as part of the HEALTHCAP project, which was approved by the Ministry of Health, Uzbekistan. Four graduate students from the Tashkent Medical Academy, for whom Uzbek and Russian were their native language, were trained as field staff in carrying out the survey.

**Figure 2:**
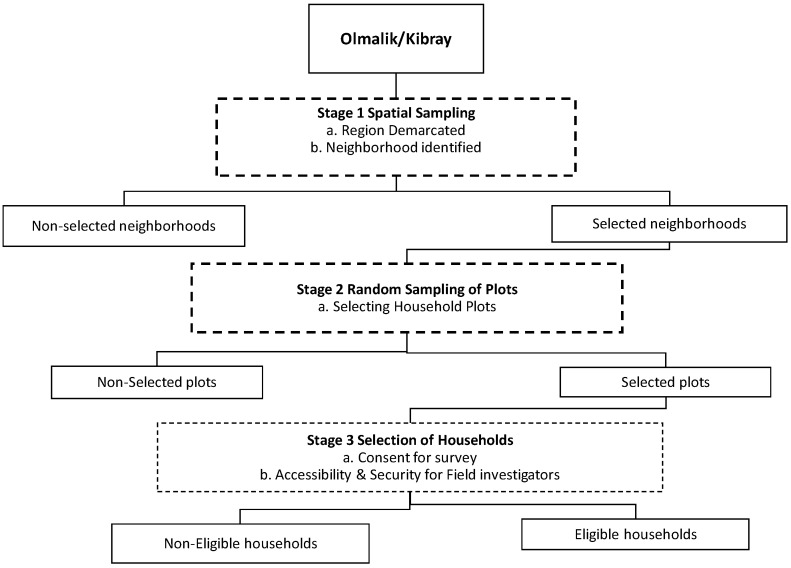
Flowchart of the surveys carried out in Olmalik & Kibray in 2015.

The survey involved three stages [30] (Figure 2). In the first stage, the region was demarcated, and neighborhoods were selected for survey. Due to the lack of official maps for the districts, the research team digitized a tourist map depicting the boundaries. In addition, the research team toured the area on foot with a local guide who is knowledgeable about boundary locations and landmarks to delineate the district boundaries. Boundaries were compiled using Google Maps and ArcGIS software (ESRI, Inc., Redlands, CA, USA) with x–y coordinates to create a simple random spatial sample (using the random point generator tool). Plots were selected for the survey in stage two. This was done by excluding non-residential uses from the sampling area, for example, water bodies, industrial land use, and construction grounds. Due to the geography of the region, the boundaries between land use areas are striking and can be discerned from satellite images (e.g., water boundaries and boundaries between residential areas, industrial areas, and natural terrain). Stage three involved finding eligible households for survey. The sampling size was determined based on resource and security constraints; the research team sought to work with available resources (four staff members, eight-week survey duration, and cellphone GPS units) and to avoid commonly known insecure areas. The sample accounted for a 20% failure rate in successfully recruiting participants.

The field staff approached selected households within the spatial grid to obtain verbal consent from the household head, in most cases the mother or elderly women in the household. Once consent was obtained, the field staff administered the survey using the ODK. A pilot survey was done with the trained field staff under the supervision of one of the authors on the use of GPS units to (1) find pre-determined sample points in the field, and (2) “mark” or record the x–y coordinates of the actual survey locations and locations of interest to the study. The field staff always travelled in pairs for security reasons. The teams made trip(s) by local taxi to the vicinity and then by foot to sample points using the provided GPS values. At each sample point, the field staff recorded a description of the household and its location. If the point was located away from a structure, such as in a field or industrial area, the surveyors were instructed to locate the nearest residence (within 100 m) to the right when facing north. The surveyors were able to locate between 10 and 25 sample points in one day.

The survey team in Olmalik needed to be fluent in Russian since the residential population is mostly of Russian ethnicity, while Uzbek fluency was needed in Kibray. As the respondents were not accustomed to certain questions in the survey, the survey team translated them into the local language and handed over a copy of the translated questionnaire, if demanded by the respondents. The survey team’s “local knowledge” aided in navigating through the field and in reaching out to the respondents. For example, in Kibray, some of the sample points were dispersed or sometimes in the process of construction, and the field assistants’ local knowledge helped to identify which buildings were residences.

Once the household was identified, the field assistants were instructed to contact a household member, 18 years of age or older, who was familiar with the health status of the household members [21]. As a safeguard against coercion and violation of privacy, the field staff did not offer any incentives or monetary benefits. The study covered 215 households in Olmalik and 210 in Kibray. After cleaning, the final dataset consisted of 207 households in Olmalik and 200 in Kibray.

Data analysis was performed using the Statistical Package for Social Sciences (SPSS for Windows, version 25.0, SPSS Inc., Chicago, IL, USA) and involved a description of the frequencies and central tendencies of the variables, as well as association tests. The Kolmogorov–Smirnov test was used to determine the normality of the data distribution. Non-parametric tests were used to test the dependent variable. Associations between the dependent variable and the independent variables were investigated using the Chi-square and Mann–Whitney test. Poisson regression was performed to test the association between the reported water transmitted disease (WTD) and the independent variables. Variables with a *p* value ≤ 0.20 in the bivariate analysis were selected to compose the multivariate model [31,32,33].

Spatial autocorrelation refers to the fact that events that occur in close proximity in geographical space are more similar than events that occur randomly across space or through time. Hence, the assumption that the samples are independent may be violated [34]. The most common test to measure whether phenomena are correlated across space is the global Moran’s I test which tests for spatial autocorrelation [35]. The Moran’s I test was implemented using R statistical software package to check for potential spatial autocorrelation. 

## 4. Results

### 4.1. Demographic and Socio-Economic Characteristics of Households

Olmalik is an old industrial township, and many households were small families or comprised of young migrant workers. On average, about three members (3.35) live in a household in this district compared to five members in Kibray (5.04). In Olmalik, most of the buildings dated to the Soviet times of the late 1970s, when apartments were constructed for industrial workers. Hence, the rooms of these apartments are limited and smaller in size, primarily suited for workers. This is reflected in the average available number of rooms per household, which was around four (3.65). However, only two rooms (1.91) were available for sleeping per household, for an average of three members per household. Being prebuilt, the houses already had major amenities (such as central heating, hot water, garage, roof and secure walls, and electricity), with 3.96 being the average score.

In Kibray, the average number of rooms was about seven (6.55) per household (an average of five members per household), which is almost double the number in Olmalik. The average number of rooms available for sleeping was four (3.92) per household in Kibray. Moreover, in Kibray, on average, about five members (4.5) of the household have access to toilets compared to relatively fewer members in Olmalik (3.2). As Kibray exhibits relatively larger household size with relatively limited access to a toilet as well as limited sleeping room which remains conducive to poor hygiene and sanitation practices. Kibray also scored relatively lower in the amenities score (3.62) (Table 1).

### 4.2. Socio-Economic Characteristics of Households

The socio-demographic characteristics are also distinctive in the two regions (Table 2). First, the majority of the households are male-headed in Olmalik (80.7) and Kibray (69.5). More than half (61.0% in Olmalik and 52.0% in Kibray) of the heads of household head have been educated up to secondary level and 13% do not have formal education. This shows the high level of literacy in the district as well as the educational requirement of the government. In Olmalik, most of the households (63.8) reported having no children in their homes, implying a worker population. About 59.4% of the households reported that their members were born in Olmalik but considered themselves to be of Russian or from other ethnicities. In Kibray, about 85.5% of the households reported being native (born in Kibray) and considered themselves as Uzbeks. The majority of the households viewed themselves as being in the middle or above in terms of their income category (93.7% in Olmalik, 93% in Kibray).

### 4.3. Housing Characteristics

Reflecting on the post-soviet industrial characteristics of the region, in Olmalik 95.7% of the households lived in old traditional housing and very few (7.7%) of the households reported having constructed or renovated their houses with their own savings (Table 2). Being native helps in investing (to apply for financing loans) in a new house and provides them with the freedom to renovate based on household member requirements [36]. This explains why in Kibray (with a high proportion of native Uzbek residents), about 56.5% of the households reported their houses as new or renovated, while about 43.5% were reportedly old. Despite having relatively new or renovated housing, only half the households reported having a sewage network in Kibray. In Olmalik, 83% of the households in the district are connected to the sewage network which reflects the industrial nature of the township of the previously built-in structure. However, based on the observations from the field, most of the sewage networks are in a dilapidated condition, resulting in open drains along the streets. Most of the households (more than 80%) in both districts reported having a piped water supply in their dwelling as their main source of drinking water. The rest of the households received water from public taps, hand pumps, and bottled water, based on the observations from the field.

### 4.4. Hygiene Indicators

Looking into the reported hygiene indicators, in Olmalik, most (78.7%) of the children defecate in diapers and potty boxes, with the rest (21.3%) not using such facilities (Table 2). The use of diapers and potty boxes helps in maintaining hygiene, if the stools are disposed of safely. Further, 43% of the households eat out frequently or occasionally in restaurants and street shops, resulting in exposure to poor quality water and food, which might expose their members to various WTDs and food-related diseases. About 70% of the households reported being aware of the usefulness of hand washing in preventing diseases. In Kibray, the use of diapers was low (32%) which implies potential unsanitary practices. About 88% of the households were aware that hand washing could prevent infectious disease. Frequently eating out in restaurants and street shops was common among 51% of the households in this district.

### 4.5. Water Transmitted Disease (WTD) Burden

The dependent variable, composite WTD score, was created to consolidate the survey questions regarding household emesis, hepatitis, enteritis, dysentery, enteric fever, and diarrhea into a single measure of household WTD burden, following the concept of the composite Gastrointestinal burden index developed by Issa et al. [37]. This was done with the goal of providing a crude estimate of water-transmitted disease. A high composite WTD score was defined as having four or more WTD episodes in a household in the past 6 months. Moderate and low composite WTD score categories were defined as less than three episodes, respectively. In these two case studies, altogether about 38.2% of the households reported the occurrence of WTDs among their family members in the last six months (January to June 2015). Of these, 61.8% of the households in Olmalik reported having more than 4 episodes. In contrast, only 21.5% in Kibray reported a high score WTD burden. This is also shown in the average number of cases reported with an Olmalik average of 3.18 while in Kibray the average is 1.71 (Table 3). 

### 4.6. Determinants of WTD Burden

Poisson regression analysis in Tashkent province showed renovated houses, a piped water supply in dwellings, amenities at home, and connection to a sewage network were significantly associated with a lower occurrence of WTD in Olmalik and Kibray (Table 4). Of various other factors studied, the household size had a 1.146-fold (*p* < 0.001) greater probability of experiencing WTD. Moreover, frequently eating out had 1.595-fold greater probability (*p* < 0.001) and occasionally eating out had 1.630-fold greater probability (*p* < 0.001) of WTD occurrences compared to never eating out.

Table 5 shows the results for the selected model using the Poisson Regression Model for each district. As there was no statistical information on the population size, the research team considered the count in the sample case study. In Olmalik, having a child less than 10 years old in the household increased the likelihood of experiencing WTD by 1.738 times (*p* < 0.001). An increase in household members (household size) would increase the probability of experiencing WTD occurrences 1.267 times more than others (*p* < 0.001). In principle, the larger the number of people living in a household, the larger the aggregate demand for sanitation and hygiene in the households which explains the household size association to the WTD occurrences. Frequency of eating out “occasionally” showed a significant positive correlation with WTD (*p* < 0.05). Having a higher score of amenities was found to be inversely related to WTD. 

In Kibray, an inverse relationship was observed for households that had higher amenities (−0.524; *p* < 0.05). An inverse relationship was seen with renovated new housing with a WTD burden (−1.518 *p* < 0.001) as well as dwellings with a piped water supply (−0.811, *p* < 0.01). This also presents similar findings to renovated housing which implies that newly built housing conditions ensure higher amenities. Like in Olmalik, eating out “frequently” also showed a significant positive correlation with WTD (*p* < 0.001). For the households in Kibray, having a death in the family in the past 6 months increased the likelihood of WTD by 2.746 times (*p* < 0.01). Two other observations were also made which were related to ethnicity and number of rooms in the house. Having more rooms in the households as well as having an ethnicity other than Uzbek showed a significant positive association with WTD (*p* < 0.05). 

## 5. Discussion

This study examines the role of socio-cultural, housing characteristics and environmental hygiene practices in influencing WTDs in Tashkent province in Uzbekistan. In Uzbekistan, surveillance based on exclusively clinical and epidemiological data is inadequate. Further, research studies on socio-economic and environmental hygiene are lacking due to restrictions and limited capacity on the part of researchers. This study attempts to fill this gap by examining two case studies in the Tashkent province: Olmalik, an industrial township, and Kibray, a rapid urbanizing region in the province. The study applied spatial grid sampling to select households and ODK to survey 407 households in the two districts. The study applied Poisson regression to identify some of the key predictors of WTDs.

The findings highlight demographic variables, housing characteristics, and environmental hygiene as key predictors in both case studies. Demographic variables such as household size and having children under 10 years of age in the household increases the occurrence of WTDs. Housing characteristics, such as old housing, no access to piped water and sewage facilities increases the chances of WTDs. It is only the availability of amenities in the household, which is a significant predictor of a decreased occurrence of WTDs. Environmental hygiene in eating places is another significant predictor for increased occurrence of WTDs, indicating poor quality of drinking water and poor amenities in the household. However, differences do exist in these case studies. In the industrial city, Olmalik, the demographic factor of having children in the household and higher household size significantly increases the WTDs. Poor housing is one of the causes of diseases in this old city with Russian-built apartments that remain in dilapidated conditions. This is reflected in the increased occurrence of diseases with decreased amenities in the household. In contrast, in the traditional society and rapidly urbanizing Kibray, demographic factors such as ethnicity (non-Uzbek) and deaths in the family have shown a significant increase in the occurrence of WTDs. Non-Uzbeks might not have good socio-economic conditions and access to health care, in an Uzbek-dominant region. This is also complemented by poor housing conditions, such as old houses, no access to piped water and lower amenities which increases the occurrence of diseases in this urbanizing case study. Our analysis reveals that the demographic factors, housing characteristics, and environmental hygiene are associated with WTD which concurs with the WHO report [38] and the systematic literature review on sanitation, hygiene, and drinking water and its association with WTD (for instance diarrhea) in low middle-income country settings [39]. The WHO findings also note that food safety aspects related to water, sanitation and hygiene need to be considered when it comes to assessing the disease burden of WTD, which in this study needs further investigation [38,40].

Given the presence of single and multiple WTDs per household, we tested (using Moran’s I) whether the same spatial autocorrelation pattern persisted in model residuals, which would indicate a spatial bias, as applied by Dormann et al. 2007; Mirta and Buliung 2014 [35,41]. The positive significant spatial autocorrelation (Moran’s I = 0.049; *p* < 0.05) indicated spatial autocorrelation of WTD, however, the value close to zero implies its minimal impact on the model results. Like other analytic methods, the current analysis has several limitations, including multicollinearity in local coefficients and multiple hypothesis testing [42,43,44] as well as spatial autocorrelation observed in field studies with design constraints [45]. Despite such limitations, random spatial sampling is still regarded as an alternative in resource limited settings [21].

This study elucidates for the first time the impact of socio-demographic and hygiene factors on WTDs in this country. Europe emerged from filthy conditions in the late nineteenth century through a sanitary revolution, which has been identified as one of the most important medical milestones in the past 150 years by the British Medical Journal [46].The integrated approach of sewage disposal and treated water piped to homes played a major role in reducing disease in Europe [7]. It is important that developing countries, like Uzbekistan, understand the integrated nature of the problem to provide basic sanitation and safe drinking water for its citizens. Policies and programs with appropriate planning, better housing regulation, integrated water supply and sanitation, and effective environmental hygiene can significantly improve public health.

## 6. Conclusions

While national and international studies have highlighted socio-cultural and environmental health issues at the national level, there has been little investigation below the national level in Central Asian countries. This paper contributes to filling this gap by examining the socio-economic, demographic, and hygiene factors influencing WTDs in the Tashkent province in Uzbekistan. Following the United Nations Development Program report [14] many international agencies have increased their investment in water supplies and sanitation to address the growing threat of infectious diseases. This has also been accelerated due to the millennium and sustainable developments goals. Although these interventions have helped households to gain access to basic drinking water and safe sanitation, the findings concur with those of Semenza et al. [9] Herbst et al. [12], and Ercumen et al. [47], highlighting that just providing drinking water and access to sanitation does not necessarily address infectious diseases. The promotion of basic health must go beyond these technocentric interventions with a focus on environmental hygiene. Such an approach should focus on hygiene in public places, regulating food, adequate regional planning and appropriate design and regulation of houses in the region.

This study was operationalized in a challenging environment which will open up new avenues for complementing macro studies with more micro-level analyses of public health in this region. Moreover, the study findings concur with the recent theory of syndemic pathways, according to which contextual and social factors create the conditions for clustering of illness and diseases which influences the WTDs. These clustering conditions increase the health burden of affected populations [48]. Further, the study highlights the need to focus on environmental hygiene, social demography, improved housing, and social behavior as key interventions rather than merely on on-site interventions such as the provision of drinking water and sanitation.

## Figures and Tables

**Table 1:** Description of household characteristics using continuous variables.

	Olmalik (*n* = 207)				Kibray (*n* = 200)			
Variable	Mean	Std. Dev	Min	Max	Mean	Std. Dev	Min	Max
Household members (size)	3.35	1.62	1	9	5.04	2.24	1	13
Average rooms per household	3.65	0.97	2	7	6.55	2.60	1	15
Number of rooms available for sleeping	1.91	0.76	1	5	3.92	2.36	1	14
Amenities in the house (Sum of amenities score)	3.96	1.38	1	6	3.62	0.98	1	6
Number of persons using a toilet	3.20	1.50	1	9	4.55	2.11	0.33	13

**Table 2:** Description of household characteristics using categorical variables.

		Olmalik (*n* = 207)	Kibray (*n* = 200)
Variable definition	Categories	% distribution	% distribution
	Socio-economic		
Household head gender	0 = female	19.3	30.5
	1 = male	80.7	69.5
Household head education	1 = Primary education	6.1	7.8
	2 = Secondary vocational education	61.0	52.0
	3 = University	12.2	17.2
	4 = others (including graduate)	11.7	9.6
	5 = unknown	9.0	13.4
Households perceiving themselves as ‘middle and above’ income category (%)	0 = ‘below middle’	6.3	7.0
	1 = ‘middle or above’	93.7	93.0
Households perceiving themselves as native (%)	0 = native	40.6	85.5
	1 = non-native	59.4	14.5
No of Households having children under 10 years	0 = no	63.8	42.5
	1 = yes	36.2	57.5
**Housing Characteristics**			
Households renovated (%)	0 = old traditional	95.7	43.5
	1 = New renovated	4.3	56.5
Households constructed or purchased the house with own savings (%)	0 = no	92.3	22.0
	1 = yes	7.7	78.0
Piped water into the dwelling is the main source of drinking water (%)	0 = no	12.6	10.5
	1 = yes	87.4	89.5
Household connected sewage network (%)	0 = No sewage network	16.4	41.0
	1 = sewage network	83.6	59.0
Household using flush Toilets (%)	0 = other including dry toilet sink hole	5.8	6.5
	1 = septic tank	22.7	25.5
	2 = flush toilet	71.0	68.0
**Hygiene Indicators of the Households**			
Children defecating in potty box and diapers (%)	0 = No	21.3	68.0
	1 = yes	78.7	32.0
Children defecating at the same place as adults	0 = no	77.3	68.5
	1 = yes	22.7	31.5
Frequency of eating out	0 = do not eat out	57.0	50.0
	1 = occasionally	22.7	37.5
	2 = frequently	20.3	12.5
Households using boiling method to make water safe to drink	0 = no	30.9	29.0
	1 = yes	69.1	71.0
Households storing water in plastic containers (%)	0 = no	59.9	58.5
	1 = yes	40.1	41.5
Household covering the drinking water containers (%)	0 = no	52.7	26.0
	1 = yes	47.3	74.0
Households using other methods (aeration, filter, freezing) to treat water (%)	0 = no	44.4	88.0
	1 = yes	55.6	12.0
Households perceiving handwashing can prevent illness (%)	0 = no	29.5	11.5
	1 = yes	70.5	88.5
Households reporting death in family in the last 5 years	0 = no	85.0	94.5
	1 = yes	15.0	5.5

**Table 3:** Occurrence of Water-transmitted diseases.

		Olmalik (*n* = 207)				Kibray (*n* = 200)			
Variable		Mean	Std. Dev	Min	Max	Mean	Std. Dev	Min	Max
Households reporting ‘no’ cases of WTDs		3.18	3.17	0	16	1.71	2.41	0	12
		**Olmalik (*n* = 207)**				**Kibray (*n* = 200)**			
	Categories	% distribution				% distribution			
Households WTD burden	0 = moderate low	38.2				78.5			
	1 = high	61.8				21.5			

Source. Field survey.

**Table 4:** The estimated proportional changes (±SE) of Selected Poisson Regression Model variables that a household would report in relation to WTD in Tashkent province (2015).

Tashkent Province (Olmalik; Kibray *n* = 407)				
Predictor	Estimate	Standard Error	*p*-Value	Exp(B)
**Intercept**	0.971	0.3105	0.042	1.344 (0.783–2.308)
**Gender of household head** **(0 = female, 1 = male)**	0.375	0.1324	0.043 *	1.401 (1.082–1.815)
**Have child in the family less than 10 years old** **(0 = No,1 = Yes)**	0.337	0.1188	0.005 **	1.447 (1.145–1.829)
**Ethnicity (0 = Uzbek, 1 = Other)**	0.059	0.1275	0.643	1.046 (0.815–1.343)
**Housing status** **(0 = old; 1 = new)**	−1.043	0.1847	0.000 **	0.338 (0.235–0.487)
**Death in the family past 6 months (0 = no 1 = yes)**	−0.026	0.1511	0.862	0.998 (0.744–1.339)
**Water source piped into dwelling (0 = no, 1 = yes)**	−0.623	0.1848	0.001 **	0.599 (0.456–0.786)
**Boiling water safe to drink** **(0 = no; 1 = yes)**	−0.034	0.1072	0.749	0.956 (0.776–1.176)
**Household size**	0.137	0.0297	0.000 **	1.233 (1.104–1.376)
**Sum of Amenities**	−0.125	0.0470	0.008 **	0.966 (0.891–1.047)
**Number of people using toilet**	−0.066	0.0548	0.228	0.934 (0.834–1.042)
**Sewage connection** **(no = 0; yes = 1)**	−0.359	0.1816	0.048 *	0.698 (0.489–0.997)
**Frequency of eating out** **(0 = never)**				
**1 = occasionally**	0.488	0.1430	0.001 **	1.595 (1.270–2.002)
**2 = frequently)**	0.467	0.1161	0.000 **	1.630 (1.231–2.157)

* significant at 0.05, ** significant at 0.01. Dependent variable: WTD composite burden.

**Table 5:** Poisson Regression Analysis with estimating the proportional changes (±SE) of household WTD burden in Olmalik and Kibray (2015).

	Olmalik (*n* = 207)				Kibray (*n* = 200)			
Predictor	Estimate	Standard Error	*p*-Value	Exp(B)	Estimate	Standard Error	*p*-Value	Exp(B)
**Intercept**	0.062	0.3972	0.877	1.132 (0.562–2.280)	−4.327	0.8598	0.000	0.005 (0.001–0.029)
**Gender of household head** **(0 = female, 1 = male)**	0.249	0.1583	0.115	1.329 (0.655–0.154)	0.288	0.2711	0.287	1.859 (1.117–3.095)
**Have child in the family less than 10 years old** **(0 = No,1 = Yes)**	0.553	0.1334	0.000 **	1.801 (1.398–2.320)	0.091	0.2836	0.749	1.166 (0.680–1.998)
**Ethnicity (0 = Uzbek, 1 = Other)**	−0.185	0.1442	0.201	0.869 (0.655–1.154)	0.900	0.4010	0.025 *	1.849 (0.836–4.085)
**Housing status (0 = old; 1 = new)**	−0.044	0.2722	0.872	0.617 (0.401–0.950)	−0.369	0.2665	0.000 **	0.446 (0.015–0.902)
**Death in the family past 6 months (0 = no 1 = yes)**	−0.188	0.1769	0.287	0.806 (0.570–1.140)	1.010	0.3471	0.004 **	2.486 (1.265–4.885)
**Water source piped into dwelling** **(0 = no, 1 = yes)**	−0.133	0.1821	0.466	0.817 (0.971–1.138)	−0.811	0.2751	0.003 **	0.444 (0.259–0.762)
**Boiling water safe to drink** **(0 = no; 1 = yes)**	−0.065	0.1296	0.617	0.714 (0.459–1.12)	0.237	0.2462	0.336	1.267 (0.782–2.053)
**Household size**	0.237	0.0393	0.000 **	1.230 (1.074–1.408)	0.013	0.0523	0.798	1.054 (0.876–1.268)
**Sum of Amenities**	−0.115	0.0488	0.019 *	0.851 (0.771–0.938)	−0.379	0.1260	0.011 *	0.460 (0.095–0.946)
**Number of people using toilet**	0.020	0.797	0.862	1.230 (0.899–1.185)	0.053	0.0912	0.560	1.063 (0.887–1.273)
**Sewage connection** **(no = 0; yes = 1)**	−0.154	0.3085	0.617	0.873 (0.617–1.236)	0.318	0.3178	0.318	1.686 (1.016–2.796)
**Frequency of eating out** **(0 = never)**								
**1 = occasionally**	0.370	0.1608	0.021 *	1.281 (0.976–1.680)	0.670	0.3680	0.069	3.030 (1.748–5.253)
**2 = frequently**	0.233	0.1395	0.095	1.466 (1.071–2.006)	1.057	0.2857	0.000 *	2.443 (1.215–4.912)

* significant at 0.05, ** significant at 0.01. Dependent variable: WTD composite burden.

## Appendix A

**Table A1:** Indicators wastewater composition JV “Maxam-Chirchik” discharged into the river Chirchik.

Indicators	2011	20012	2013	2014	2015
pH	6.47	6.92	6.74	6.12	6.43
Suspended substances, mg/L	475.9	488.8	492.2	527.7	512.9
Chlorides, mg/L	265.5	294.4	276.7	292.2	266.7
Sulphates, mg/L	624.4	697.7	677.9	687.8	692.7
Dry residue, mg/L	2674	2927	2889	2764	2991
Ammonia, mg/L	122.8	102.0	104.9	74.2	83.9
Nitrite, mg/L	131.3	135.1	133.9	127.7	90.7
Nitrates. mg/L	1951	1950	1883	1903	1973
BOD5, mg02/L	88.7	93.3	77.4	76.3	66.0
COD, mg02/L	552.2	511.9	485.0	455	429.2
Petroleum products	17.9	14.2	12.3	13.4	13.6
Cyclohexane	3.67	3.74	3.92	3.12	3.06
